# A Hybrid Color Space for Skin Detection Using Genetic Algorithm Heuristic Search and Principal Component Analysis Technique

**DOI:** 10.1371/journal.pone.0134828

**Published:** 2015-08-12

**Authors:** Mahdi Maktabdar Oghaz, Mohd Aizaini Maarof, Anazida Zainal, Mohd Foad Rohani, S. Hadi Yaghoubyan

**Affiliations:** Faculty of Computing, Universiti Teknologi Malaysia, Johor Bahru, Malaysia; CSIR-Institute of Microbial Technology, INDIA

## Abstract

Color is one of the most prominent features of an image and used in many skin and face detection applications. Color space transformation is widely used by researchers to improve face and skin detection performance. Despite the substantial research efforts in this area, choosing a proper color space in terms of skin and face classification performance which can address issues like illumination variations, various camera characteristics and diversity in skin color tones has remained an open issue. This research proposes a new three-dimensional hybrid color space termed *SKN* by employing the Genetic Algorithm heuristic and Principal Component Analysis to find the optimal representation of human skin color in over seventeen existing color spaces. Genetic Algorithm heuristic is used to find the optimal color component combination setup in terms of skin detection accuracy while the Principal Component Analysis projects the optimal Genetic Algorithm solution to a less complex dimension. Pixel wise skin detection was used to evaluate the performance of the proposed color space. We have employed four classifiers including Random Forest, Naïve Bayes, Support Vector Machine and Multilayer Perceptron in order to generate the human skin color predictive model. The proposed color space was compared to some existing color spaces and shows superior results in terms of pixel-wise skin detection accuracy. Experimental results show that by using Random Forest classifier, the proposed *SKN* color space obtained an average F-score and True Positive Rate of 0.953 and False Positive Rate of 0.0482 which outperformed the existing color spaces in terms of pixel wise skin detection accuracy. The results also indicate that among the classifiers used in this study, Random Forest is the most suitable classifier for pixel wise skin detection applications.

## Introduction

Colors are an intrinsic property of every object, caused by interaction of the light spectrum with the eye’s light receptor cells [1]. In image processing and computer vision, colors are usually described mathematically as a set of numbers termed color space [2]. There are various different color spaces, each created for a specific purpose. RGB is the most common color space in digital image world [3,4,1]. Any other color space can be obtained from a linear or nonlinear transformation of RGB color space. Color space transformation is widely used by researchers to improve skin and face detection performance. Majority of the existing skin detection techniques include a color space transformation which is aimed to increase the separability between skin and non-skin colors[5], reduce the average correlation among different color components of color space [4], separate the Intensity and Chrominance components [5] and increase the likeness and unity of the skin tones of different human ethnic groups [6].

Despite the substantial research efforts in this area, choosing an optimal color space in terms of skin and face classification has remained an open issue. During the last decade, several conventional color spaces such as RGB, nRGB, YCbCr, HSI, HSV, CIEXYZ, YUV, YPbPr, TSL and CIELAB have been applied to skin detection. Each has its own advantages and drawbacks. Some researchers including [7,8,9] used hybrid color space as an alternative to conventional color spaces for skin detection applications. Hybrid color spaces consist of combination of color components from different color spaces. The notion of hybrid color space has opened up a new dimension of research in choosing a proper color space for skin and face classification purposes.

This research aims to propose a new hybrid color space to improve skin detection accuracy. The proposed hybrid color space termed *SKN* uses Genetic Algorithm (GA) heuristic which finds the optimal color component combination setup in terms of skin detection accuracy from the seventeen existing color spaces including HSI, HSV, LAB, LUV, nRGB, RGB, TSL, XYZ, YCbCr, YCgCr, YES, YIQ, YPbPr, YUV,RIQ, YQCr and i1i2i3. This research also uses Principal Component Analysis (PCA) to shrink the GA optimal solution to a lower dimension. The proposed 3-dimenisonal color space deploys three significant Principal Components of GA optimal solution as its components. In order to evaluate and compare the performance of the proposed color space versus the existing ones, we have used pixel wise skin detection methods. Since pixel wise skin detection methods are relatively simple and they are only rely on color information as the main discriminative feature, they can reflect the pure impact of the color space better than some complex techniques which might involve many different dependent factors to the detection performance.

Experimental results and comparison analysis shows that the proposed hybrid color space outperformed some of the existing color spaces in terms of pixel wise skin detection accuracy. In order to evaluate the performance of the proposed color space, Random Forest (RF), Naïve Bayes (NB), Support Vector Machine (SVM) and Multilayer Perceptron (MLP) classifiers were used to generate the human skin color predictive model. The experiments were carried out on three different datasets, namely: Dataset A which includes hand gesture images from HGR dataset, Dataset B which consists of images from ECU face and skin detection dataset and Dataset C which includes facial images from AR and color FERET datasets. Qualitative and quantitative analysis and comparison of results indicate the dominance of the proposed hybrid color space over the existing ones in terms of pixel wise skin detection. The paper is organized as follows: section 2 discusses the related works, section 3 presents the methodology, section 4 describes the results and analysis and finally section 5 concludes the paper.

## Related Works

Although few a researchers believe that color space transformation does not make any significant improvement in skin detection [5,10], most agree on its importance in skin and face detection performance. Numerous studies have used color space transformation to improve pixel wise skin detection accuracy. In terms of the color space they have used, these studies can be categorized into three groups including conventional color spaces, domain specific color spaces and hybrid color spaces.

The majority of the researchers have been focused on conventional color space transformation to enhance skin and face detection performance. Simplicity of the implementation might be the main reason for the popularity of conventional color spaces. Many researchers including Bergasa et al. [11], Brown at al. [12], Oliver et al. [13], Wang and Sang [14], Yang and Waibel [15] have employed nRGB color space for skin and face detection purposes. They believe that by using nRGB color space, skin color cluster domain has relatively lower variance under different illumination conditions compared to RGB color space. CIEXYZ is another commonly used conventional color space which was designed based on the response curves of the three color receptors of human eyes. Strong correlations among CIEXYZ components make it an unfavorable color space for any image segmentation application including skin and face detection; however some researchers including Chen and Chiang [16], Brown et al. [12] and Gonzalez et al. [17] used this color space for skin detection purposes. HSV and HSI are two popular cylindrical color space that have been employed by many researchers including Khan et al. [18], Zarit et al. [6], Gonzalez et al. [17], Juang and Shiu [19], Kim et al.[20], Singh et al. [21], Zainuddin et al.[22], Oliveira and Conci [23], Kovac et al. [24] for skin and face detection aims. Since these color spaces separate the illumination and chrominance, they might be suitable for skin and face detection under uncontrolled illumination condition. YCbCr is another conventional color space which is mainly designed as a digital approach to handle video information in color television transmission systems. It is widely used by many researchers such as Gonzalez et al. [17], Schmugge et al. [25], Khan et al. [18], Subban and Mishra [26], Zarit et al. [6], Aibinu et al. [27], Chai and Bouzerdoum [28] to improve skin and face detection accuracy. Xu et al. [29] used the quaternion number to represent the three components of a color pixel, then employed linear discriminant analysis algorithm to transform the quaternion vector into a lower dimension. They believe that this method can obtain a very high accuracy for color face recognition.

Since conventional color spaces are not primarily designed to deal with skin and face detection issues, some researchers have gone further than the conventional color spaces and designed domain specific color spaces which are specifically aimed at enhancing the skin and face detection performance. For example, De dios and Garcia [30,31] proposed YCgCr color space to enhance face detection performance. This color space was later adopted by many researchers including Subban and Mishra [26], Ghazali et al. [32], Zhang and Shi [33], Ghazali and Hawari [34] for the same purpose. In addition to YCgCr, TSL developed by Terrillon et al. [35,36] is another domain specific color space which aimed to improve skin and face detection performance. This color space has been used by other researchers including Brown et al. [12] Tomaz et al. [37] Vezhnevets et al. [38] for skin and face detection applications.

Despite the advantages that a domain specific color space might bring to skin and face detection, designing a new domain specific color space is a challenging and complex process and requires many adjustments and considerations. This prompted researchers to consider hybrid color spaces which provide some of the benefits of domain specific color spaces at relatively lower operational complexity. Hybrid color spaces are formed by putting different components of conventional color spaces together. Many researchers used hybrid color spaces for skin and face detection applications. Shih and Liu in [8] proposed a hybrid color space based on individual or combination of color components in YIQ and YCbCr color spaces. They showed that YQCr outperformed other component combinations in terms of skin detection. Another research by Shih and Liu [39] compared different combinations of seven conventional color spaces and concluded that YV in the YUV color space and YI in the YIQ color space improve face detection performance. In another research Liu and Liu in [40] proposed a new hybrid color space RIQ, which combines the “R” component of the RGB color space and “I” and “Q” chromatic components of the YIQ color space for improving face recognition performance. Despite the efforts of researchers to initiate and adopt hybrid color spaces for skin and face detection applications, lack of a comprehensive hybrid color space which involves a wider range of existing color spaces is tangible. We believe that such a solution can address common skin detection problems.

## Methodology

**Fig 1:**
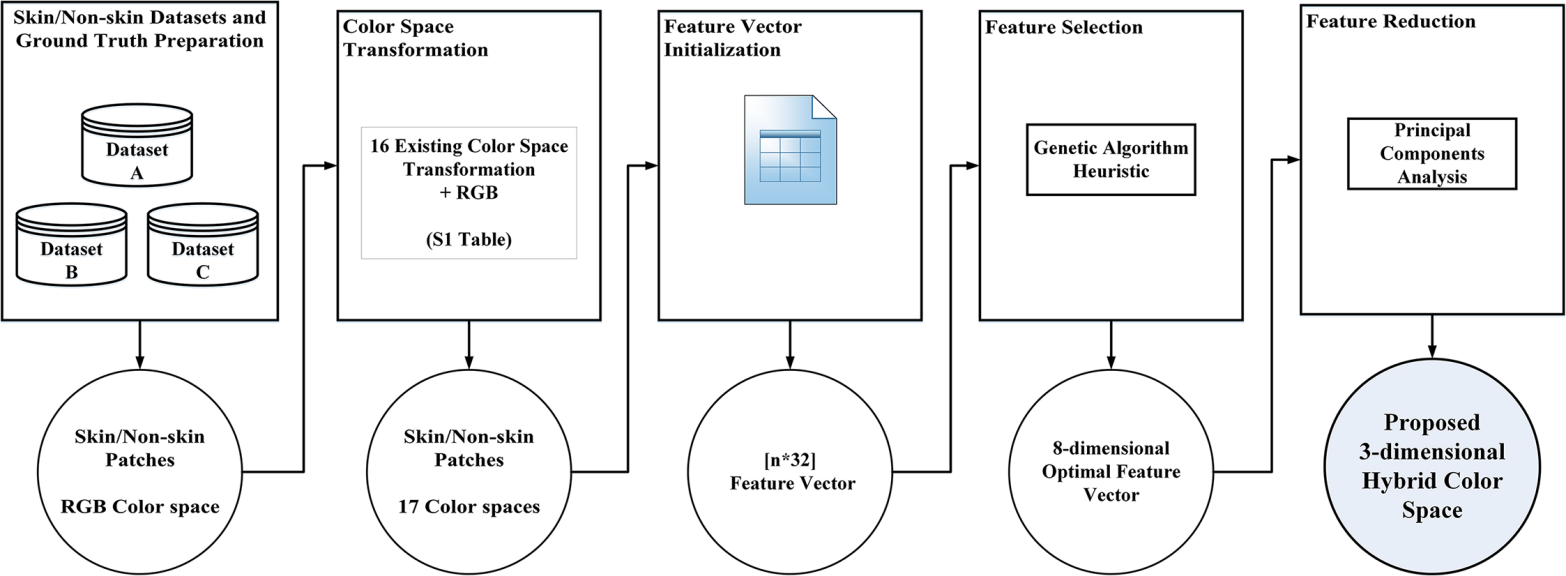
Block diagram of the steps that forms the proposed hybrid color space for skin detection.

Hybrid color spaces are formed by integration of color components from different existing color spaces. The key point in forming a hybrid color space for skin detection is to find a combination setup of color components which improves the ability to classify human skin. In this study seventeen existing color spaces including HSI, HSV, LAB, LUV, nRGB, RGB, TSL, XYZ, YCbCr, YCgCr, YES, YIQ, YPbPr, YUV, i1i2i3, RIQ and YQCr are forming the foundation of our proposed hybrid color space. These color spaces which are frequently used for face and skin detection applications contain a total of 32 unique color components. Any combination setup of these color components can be a potential solution to this study. Since evaluation of each combination setup takes a considerably long time, this research uses GA Heuristic and PCA to find the optimal color component combination setup for skin detection in a relatively faster and more intelligent fashion compared to conventional techniques such as exhaustive or greedy search. Fig 1 shows the block diagram of the steps that forms the proposed hybrid color space.

According to Fig 1 the proposed color space is formed through five steps *including Skin/Non-skin Datasets and Ground Truth Preparation*, *Color Space Transformation*, *Feature Vector Initialization*, *Feature Selection* and *Feature Reduction*. The following section describes each step in more details:

### Skin/Non-skin Datasets and Ground Truth Preparation

Datasets of skin/Non skin provide an accurate estimation of skin and Non-skin color cluster which is essential to form our hybrid color space. These datasets alongside the corresponding Ground Truth are also used for training, testing and evaluating purposes in classification stage. The experiments in this study where carried out for three datasets namely, Dataset A, Dataset B and Dataset C.

**Dataset A,** termed HGR, consists of 899 hand images from 12 individuals which have been used for hand gesture recognition purposes. The image dimensions vary from 174x131 up to 640x480. All images are captured in uncontrolled background and lighting condition. This database was developed by Kawulok et al. [41] as a part of a hand detection and pose estimation project. The images from HGR dataset are associated with ground truth skin binary mask indicating the skin regions.

**Dataset B** consists of 400 images from ECU face and skin detection dataset developed by Phung et al. [42]. These images where chosen as they ensure diversity in terms of background scenes, lighting conditions, and face and skin types. The ground-truth images were meticulously prepared by manually segmenting the face and skin regions. For more information on ECU dataset please refer to [42].

**Dataset C** consists of a collection of 1118 facial RGB images collected from AR and COLOR FERET datasets. The AR dataset is frequently used for face detection purposes and contains more than 4000 frontal face images. Meanwhile, the COLOR FERET dataset developed by Defense Advanced Research Agency (DARPA) in 2003 contains 2400 facial images from over 800 individuals. Dataset C contains facial images from different individuals, genders, ethnics, lighting conditions and camera settings. The images from AR and FERET dataset are associated with ground truth skin binary mask indicating the skin regions. For more information on AR and FERET datasets please refer to [43,44]. A brief summary of datasets statistics is given in Table 1.

**Table 1:** Dataset Statistics Summary.

	Dataset A	Dataset B	Dataset C
**Total Number of Images**	899	400	1118
**Contents**	Hand Gesture	Face and Skin	Facial
**Total number of pixels**	~ 190 million	~ 41 million	~ 1344 million
**Total number of Skin pixels**	~ 38.7 million	~ 7 million	~ 386 million
**Total number of non-skin pixels**	~ 150 million	~ 34 million	~ 958 million
**Ground Truth Techniques**	Manually	Manually	Manually
**Source**	HGR Dataset	ECU dataset	AR and COLOR FERET datasets
**Lighting**	Indoor Uncontrolled	Indoor/outdoor Uncontrolled	Indoor Uncontrolled
**Gender**	Male / Female	Male / Female	Male/ Female
**Skin Type**	Light	Light / Dark	Light / Dark
**Color Space**	RGB	RGB	RGB

Please refer to the guidelines on (S1 File) to access the datasets that we used in this study.

### Color Space Transformation

In this stage, all images from our datasets undergo 16 color space transformations including HSI, HSV, LAB, LUV, nRGB, TSL, XYZ, YCbCr, YCgCr, YES, YIQ, YPbPr, YUV, i1i2i3, RIQ and YQCr. The color space transformed images alongside the images in RGB color space form 17 different representations of Skin/Non-skin color cluster distribution which initiates the feature vector of this study. The color space transformation formulas can be found in S1 Table.

### Feature Vector Initialization

Feature Vector Initialization is the process of transforming the visual image data into vector of features (color components) in order to perform optimization and data mining operations in the further stages. To initiate the feature vector of this study, all 3-dimansional color space transformed images from the previous step alongside the RGB images are reshaped into 3-column matrices where the rows represent pixel values and columns resemble the color components. Horizontal concatenation of these matrices yields a [n*32] matrix (redundant color component has been removed) where *‘n’* is the total number of pixels and 32 denotes the number of unique color components in 17 color spaces. These 32 vectors form color components which are initiating the feature vector of this study including: H_HSI_, S_HSI_, I_HSI_, L_LAB_, A_LAB_, B_LAB_, U_LUV_, V_LUV_, R_nRGB_, G_nRGB_, B_nRGB_, R_RGB_, G_RGB_, B_RGB_, S_TSL_, L_TSL_, X_XYZ_, Y_XYZ_, Z_XYZ_, Y_YCBCR_, CB_YCBCR_, CR_YCBCR_, CG_YCGCR_, CR_YCGCR_, E_YES_, S_YES_, Q_YIQ_, PB_YPBPR_, PR_YPBPR_, i1_i1i2i3_, i2_i1i2i3_, i3_i1i2i3_. The associated ground truth skin binary mask of each image also reshaped into a vector which indicates the corresponding class of each pixel. We have “*skin*” and “*non-skin*” classes denoted by “1” and “0” respectively.

### Feature Selection (Genetic Algorithm Heuristic)

**Fig 2:**
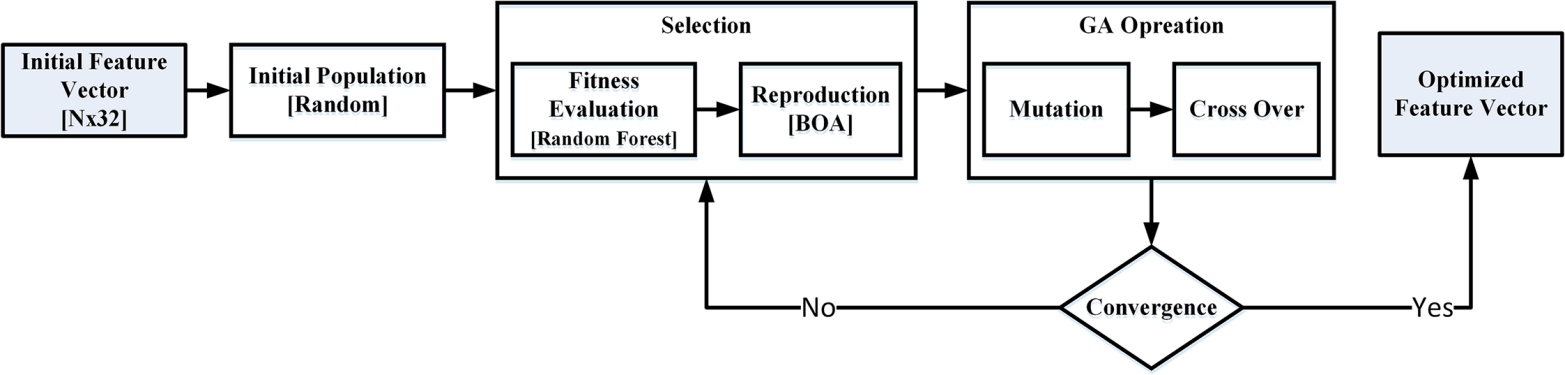
Block diagram of feature selection stage using Genetic Algorithm.

This stage is aimed to find the optimal subset of features (color component combination setup) from the initial 32 dimensional feature vector which improves the accuracy of the skin detection predictive model. This usually can be achieved by removing the less discriminative, correlated, noisy or redundant features from the feature vector. A feature vector of 32 features generates C_(32,3)_ = 4960 different combination setups. Considering such a huge search space and the time required to evaluate each combination setup, we have used GA Heuristic to find the optimal combination setup of features (color components) in order to maximize the accuracy of the pixel wise skin detection. Fig 2 shows the block diagram of the feature selection stage using GA. In the following section we investigate the impact of *population size*, *cross over*, *mutation*, *fitness function* and *reproduction technique* on GA performance.

Initial population which is generated randomly consists of individuals (candidate solutions) each contains a random set of features (color components). Random generation of initial population allows the entire search space to contribute in forming the optimal solution.

**Fig 3:**
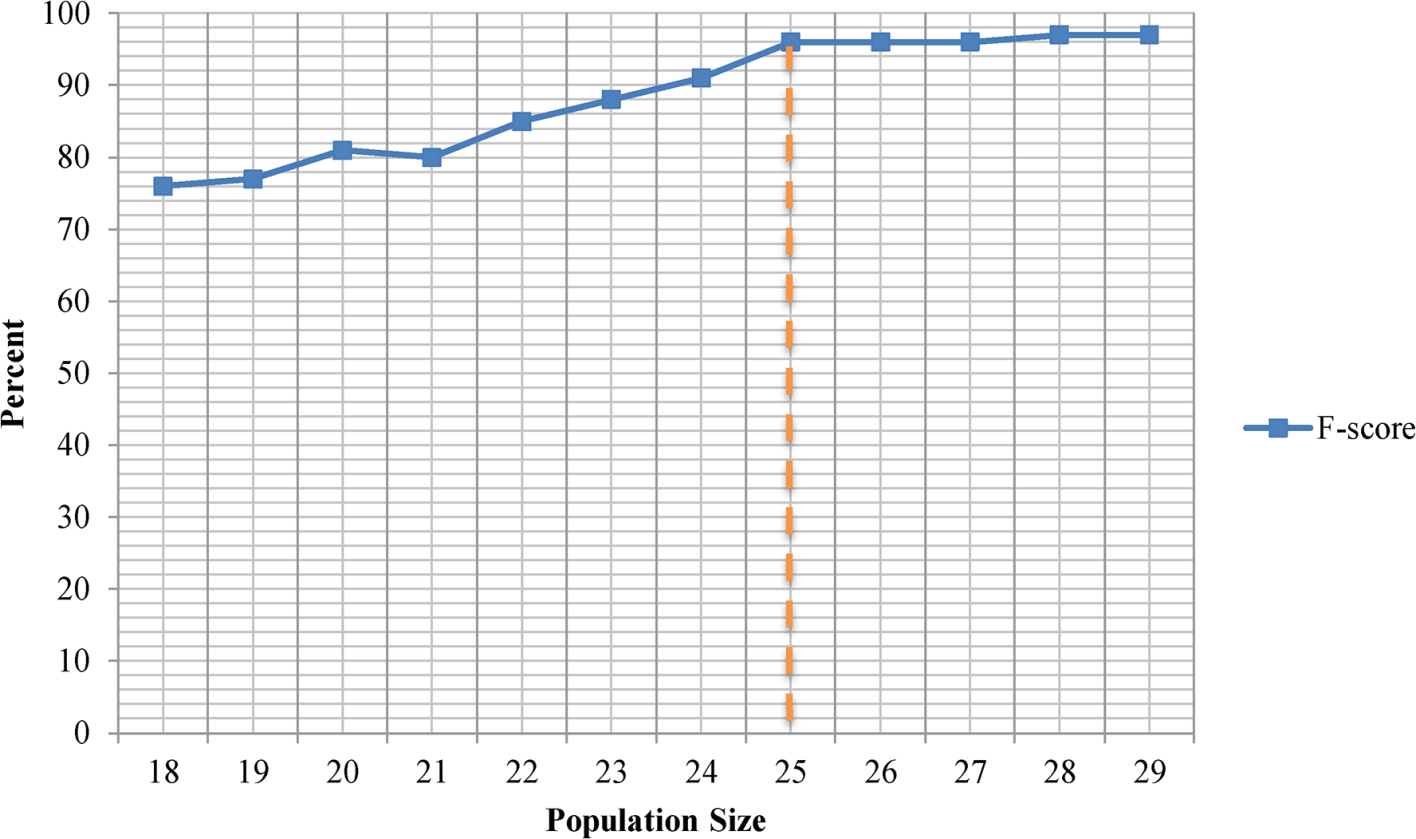
F-score of the Genetic Algorithm optimal solution under different population sizes ranging from 18 to 30 individuals. Random Forest classifier used to generate the skin detection predictive model.

Proper population size is very dependent on the nature of the problem. To determine the right population size, we have measured the F-score of the GA optimal solution under different population sizes ranging from 18 to 30 individuals as shown in Fig 3. We have noticed that smaller population sizes decrease the computational complexity of the operations but may increase the chance of premature convergence and trap the systems into local maxima. On the other hand larger population sizes increase the computational load and retards the convergence while it does not always leads to a better solution. In our experiments, we found that at population size of 25 individuals, GA solution delivers the optimum skin detection results. At population sizes smaller than 25, GA is frequently trapped in local maxima while population sizes larger than 25 only increased the computational complexity of the operations however they nearly converged to the identical solution.

This study used Random Forest Classifier as GA fitness function. We used the same parameters for Random Forest as Khan et al. in [45,18]. The F-score value is used as the primary evaluation measure (figure of merit). In every generation Random Forest measures the fitness of each individual in terms of skin detection accuracy. 10 fold stratified cross validation was used to validate the accuracy of each individual. Bayesian Optimization Algorithm (BOA) is used in order to generate the successive generations. After each iteration, individuals with higher fitness form a Bayesian network which partially initiates the successive generation’s population. The rest of the population will be generated randomly to replace the unfit individuals.

**Fig 4:**
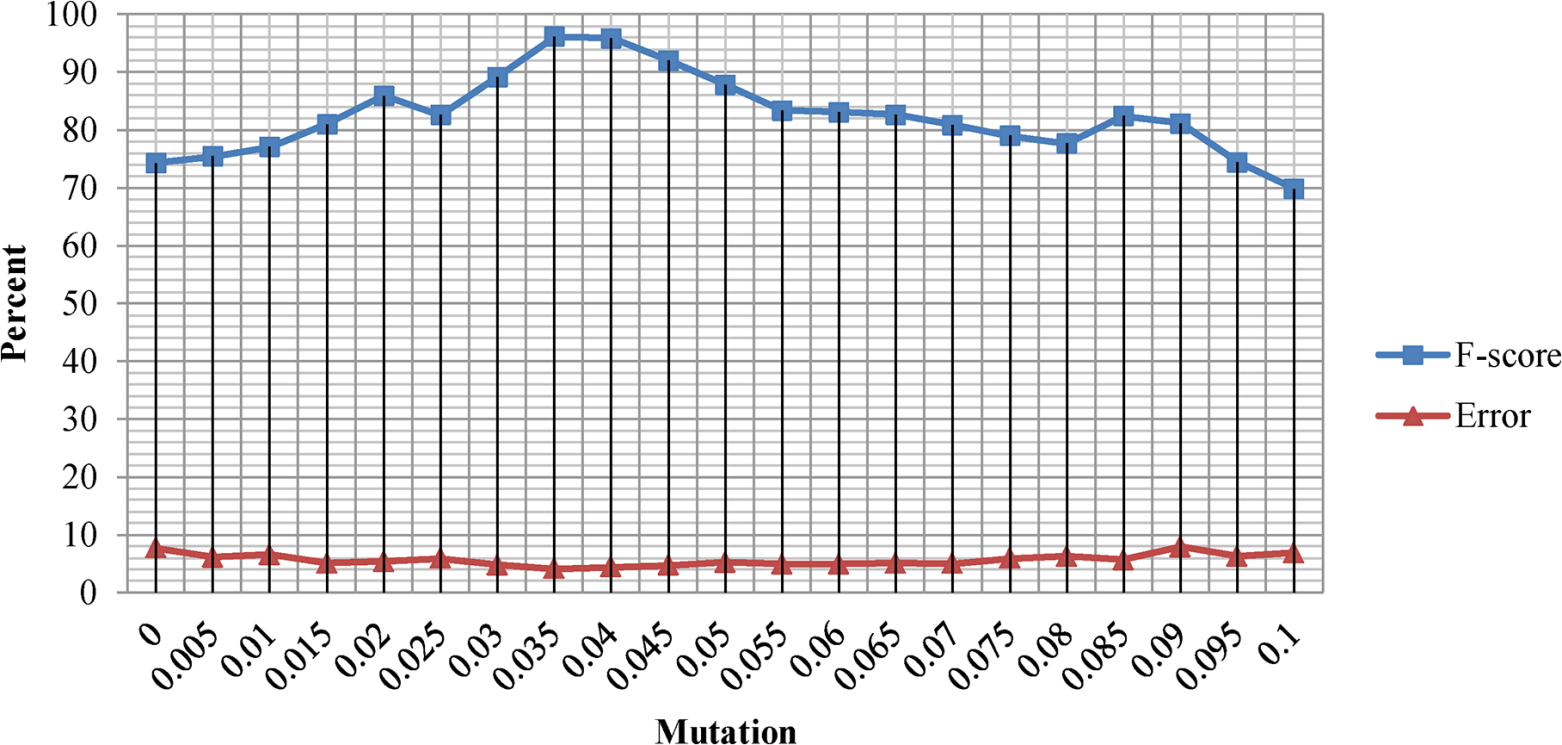
F-score and Error rate of the Genetic Algorithm optimal solution under mutation probabilities ranging from 0 to 0.1.

**Fig 5:**
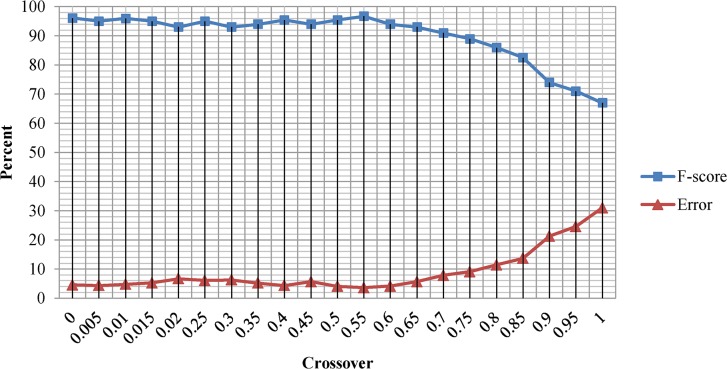
F-score and Error rate of the Genetic Algorithm optimal solution under crossover probabilities ranging from 0 to 1.

The Mutation and crossover probabilities were empirically set to 0.035 and 0.55 respectively. We observed that higher mutation probabilities prevent the population from converging to global maxima while lower mutation probabilities lead to premature convergence and trap the systems into local maxima. Experiments also revealed that crossover probability higher than 0.55 decrease the accuracy of the GA optimal solution while lower crossover probabilities retard the convergence. Fig 4 shows the F-score and Error rate of the GA optimal solution over different mutation probabilities ranging from 0 to 0.1. According to Fig 4, the average Error rate of the GA optimal solution remains relatively consistent over the entire mutation probability range. On the other hand, the F-score value has relatively inconsistent behavior over the entire range of mutation probabilities. Fig 5 shows the F-score and Error rate of the GA optimal solution over different crossover probabilities ranging from 0 to 1. According to Fig 5, the average F-score and Error rates of GA optimal solution remains relatively consistent in crossover probabilities ranging from 0 to 0.60, while a slight drop in F-score and Error rates was observed as crossover probability rises from 0.6 to 1.

**Fig 6:**
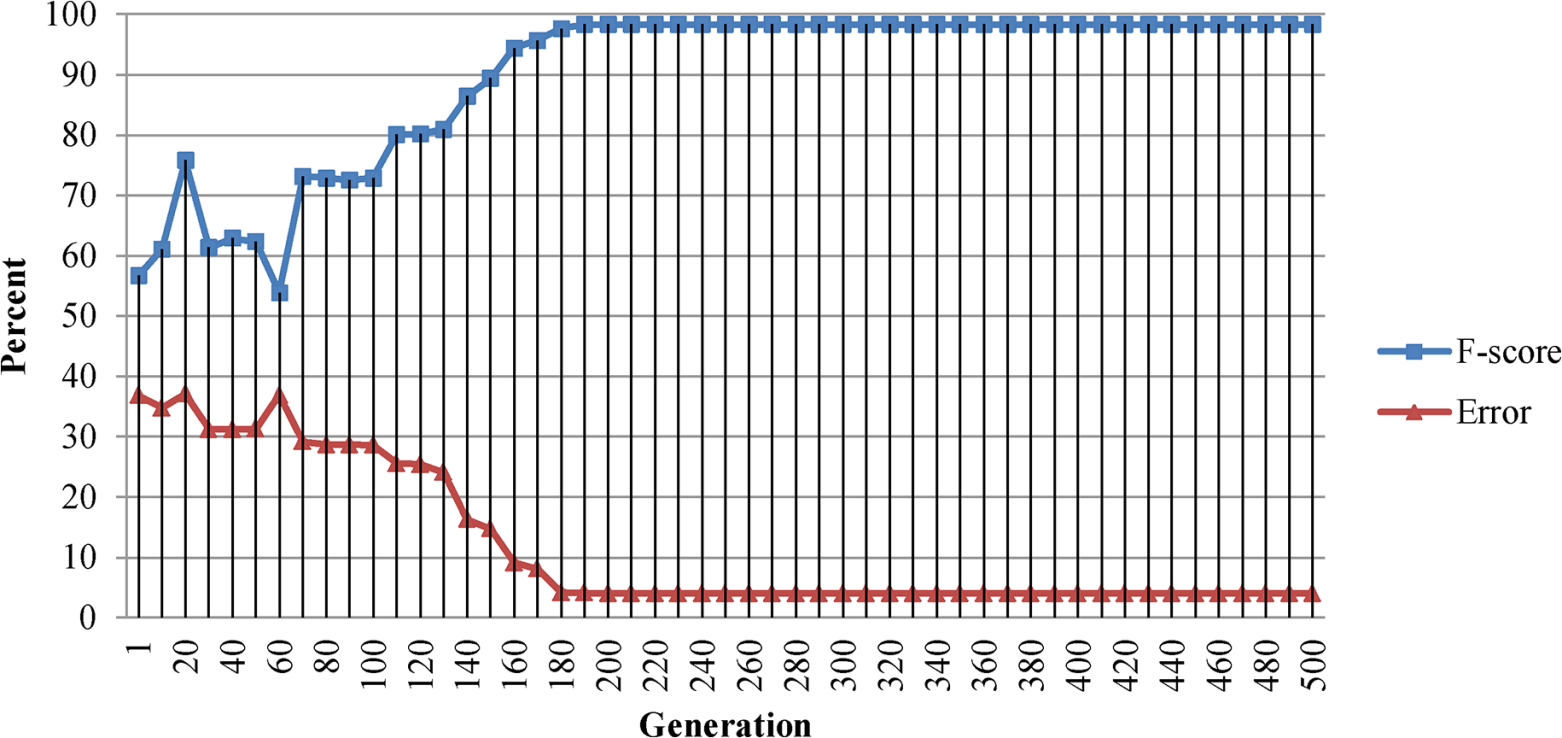
F-score and Error rate of the Genetic Algorithm optimal solution over generations. Convergence observed after around 180 generations.

GA generational process is repeated until a termination condition has been satisfied. In this study maximum number of iterations is set to 500 generations; however GA converged to the global maxima after 180 generations. Fig 6 displays the F-score and Error rate of the optimal solution over generations.

GA candidate optimal solution remained unchanged from 180 to 500 generations which indicates that the GA converged to its global maxima. Results indicate that combination setup of 8 color components including V_HSV_, G_nRGB_, B_RGB_, Y_XYZ_, Z_XYZ_, Y_YCbCr_, S_YES_ and i3_i1i2i3_ which delivers F-score of 0.983 is the optimal color component combination setup for skin detection.

### Feature Reduction (Principal Component Analysis)

**Fig 7:**
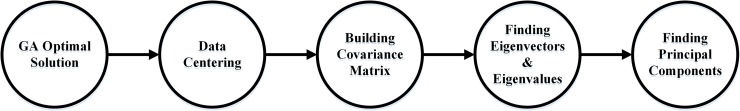
Block Diagram of the Principal Component Analysis.

Using the 8-dimensional GA optimal solution as a color space might deliver very high skin detection accuracy, but this 8-dimensional solution is computationally expensive when it is compared with the existing 3-dimensional color spaces. In order to counter this problem, we have employed the PCA technique in order to project the 8-dimensional GA optimal solution to its Principal Components, to reduce its dimensions with minimum loss in data variance. More information on PCA can be found at [46,47]. Fig 7 shows the steps to find the Principal Components of the GA optimal solution.

In the first step, 8-dimensional GA optimal solution goes through a data centering process to ensure that its 1^st^ Principal Component represents the maximum data variance direction. We have used Mean Subtraction technique for data centering as formula in (1):

$$\text{C}=\sum_{\text{i}=1}^{\text{n}}\left({\text{x}}_{\;\text{i}}-\bar{\text{x}}\right)$$ (1)

where $\bar{x}$ denotes color component mean, *x* enotes the pixel (color) value, *n* is total number of instances and *C* represents the centralized color value. The next step involves finding the Covariance matrix of the centered 8-dimensional GA optimal solution. Covariance matrix is an [8x8] matrix whose element in *i*, *j* position denotes the covariance between *i* ^*th*^ and *j* ^*th*^ color components in the GA optimal solution. Covariance matrix “*cov*” is defined by the formula in (2).

$$\text{cov}=\left[\begin{array}{llll} \sigma_{11} & \sigma_{12} & \ldots & \sigma_{1\text{n}}\\ \sigma_{21} & \sigma_{22} & \ldots & \sigma_{2\text{n}}\\ \;\vdots & \;\vdots & \ddots & \;\vdots \\ \sigma_{\text{n}1} & \sigma_{\text{n}2} & \ldots & \sigma_{\text{nn}} \end{array}\right]\;,\;\sigma_{\text{ij}}=\frac{\sum_{1}^{\text{n}}\left(\text{i}-\bar{\text{i}}\right)\left(\text{j}-\bar{\text{j}}\right)}{\text{n}-1}$$ (2)

where *“n”* denotes the total number of instances (pixels), *σ*_*ij*_ denotes the covariance between *i* ^*th*^ and *j* ^*th*^ color components in GA optimal solution.

**Fig 8:**
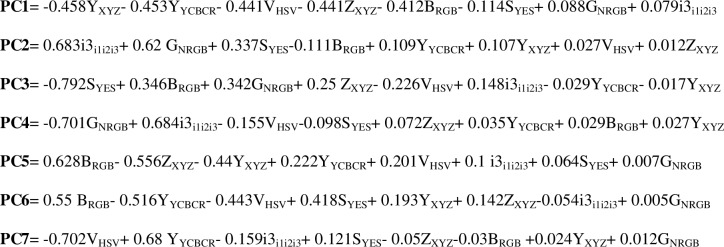
Seven most significant Principal Components of GA optimal solution.

The next step involves with finding the Eigenvectors and Eigenvalues of the covariance matrix. For square covariance matrix *Cov*, scalars *λ* and vectors *v*_*n×1*_ *≠ 0* which satisfy *Cov.v* = *λv* are called eigenvalues and eigenvectors of *cov*, respectively. The Eigenvectors are formed as an [8x8] matrix whose columns represent the coefficients of the Principal Components of the GA optimal solution. Eigenvalues are also formed as an [8x8] diagonal matrix. Eigenvector with largest corresponding eigenvalue is the first Principal Component. Similarly, the second and the subsequent principal components can be found accordingly. Principal Components are expressed through linear combination (sum of product) of the color components and the eigenvector coefficients. Fig 8 shows the seven most significant Principal Components of GA optimal solution. According to our experiments these seven Principal Components are able to cover up to 99.9 percent of the GA optimal solution variance.

**Fig 9:**
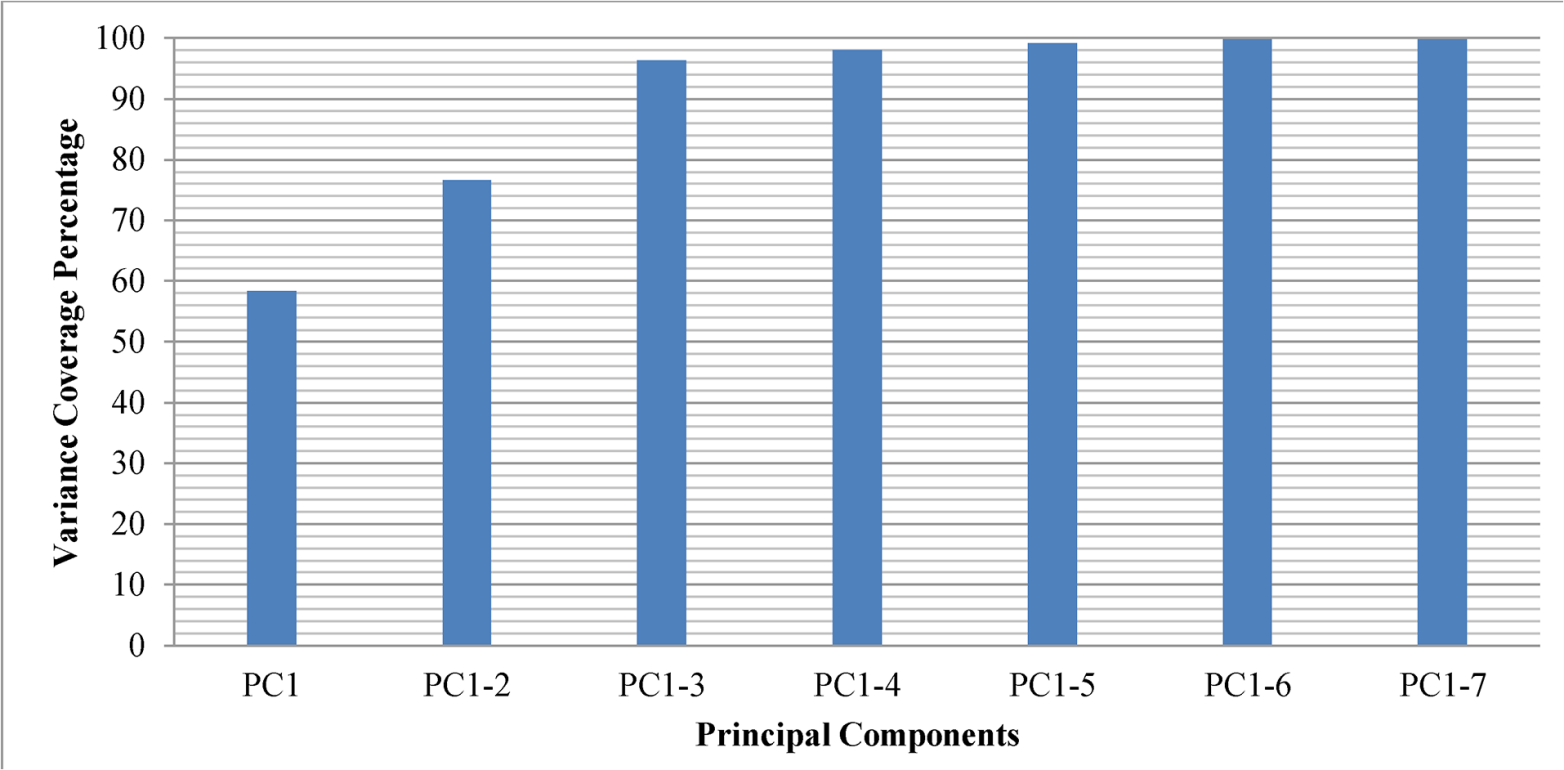
Cumulative variance of GA optimal solution explained by Principal Components.

Fig 9 shows the cumulative variance that is explained by principal components. According to our experiments the first Principal Component can cover up to 58.4 percent of the GA optimal solution variance. The first two Principal Components cover 76.7 percent of the variance and top three Principal Components hold up to 96.3 percent of the variance. The subsequent Principal Components only cover a small remaining fraction of the GA optimal solution variance. Using top three Principal Components, we are able to retrieve more than 96 percent of GA optimal solution variance which is fair enough for the purpose of this study.

These three Principal Components which closely resemble the 32 color components in initial feature vector are used to create our proposed 3-dimensionl hybrid color space. The proposed color space is termed “*SKN*” (taken from Skin) where “*S*” resembles the 1^st^ Principal Component, “*K*” denotes the 2^nd^ Principal Component and “*N*” indicates the 3^rd^ Principal Component. Considering the PC1, PC2 and PC3 equations in Fig 8 and color space conversion formulas in S1 Table, the proposed 3-dimensional hybrid color space can be reformulated using the Algebraic Simplification as function of R, G and B as shown in formula (3), (4) and (5):

$$S=PC1=0.088\frac{\text{G}}{\text{R}+\text{G}+\text{B}}-58.89\text{G}-30.014\text{R}-11.952\text{B}-7.24$$ (3)

$$K=PC2=2.122\;\text{B}+14.859\;\text{G}+6.921\text{R}+0.62\frac{\text{G}}{\text{R}+\text{G}+\text{B}}+1.744$$ (4)

$$N=PC3=0.342\frac{\text{G}}{\text{R}+\text{G}+\text{B}}-3.698\text{G}-2.25\text{R}-0.103\text{B}-0.464$$ (5)

In the next section we evaluate the impact of proposed color space on skin detection performance and compare it with existing color spaces.

## Experimental Results and Analysis

A set of qualitative and quantitative experiments was performed to analyze and evaluate the effects of the proposed color space on skin detection accuracy. This section begins with a brief description of the experimental setup and evaluation metrics, then presents the qualitative and quantitative assessment of the proposed color space in terms of pixel wise skin detection accuracy. Finally, we present a comparison between the proposed color space and the existing ones in terms of pixel wise skin detection performance.

### Experimental Setup

We employed four classifiers including Naïve Bayes, Random Forest, Support Vector Machine and Multi-layer Perceptron for pixel wise skin detection. These algorithms are the commonly preferable choices for classification problems and are used by many researchers.

**Random Forest** Decision Tree classifier introduced by Breiman [48] is used in many image classification applications like face detection and hand gesture analysis. Random Forest Decision Tree classifier uses bootstrap aggregation technique on ensemble of decision trees for classification purpose. Random Forest benefits include high generalization accuracy and quick training time. The number of decision trees is the most important factor in Random Forest classifier. In this study, maximum accuracy in Random Forest classifier was observed when 15 trees were grown. Apart from the number of trees, this study uses the same parameters for Random Forest as Khan et al. in [45].

**Multilayer Perceptron (MLP)** is a feed-forward neural network classifier which uses backpropagation supervised learning technique to train the network. A comprehensive introduction to MLP can be found in [49]. Many researchers including [18,42,50,51,52] used MLP for skin segmentation purposes. In this study we have used a network of five layers including the input layer which receives the input data from three color components in color space, three hidden layers and the output layer which designate the skin and non-skin classes. The number of neurons in each hidden layer yields through the average number of the input and output variables. The layers in MLP are connected in a feed-forward topology by weighted connections through which each neuron receives inputs, and after generating an output, broadcasts it to neurons in the next layer.

**Naïve Bayes** is a probabilistic classifier based on Bayes theorem which assigns a new observation to the most probable class. Since human skin color does not really fit into normal (Gaussian) distribution, we used kernel smoothing density distribution to estimate the probability of features. Naïve Bayes is basically designed for use when features are independent of one another. In the training stage, Naïve Bayes estimates the parameters of a probability distribution, assuming features are conditionally independent given the class. In the testing stage it computes the posterior probability of that test sample belonging to each class. The method then classifies the test sample according the largest posterior probability. This classifier is incomparably fast compare with other classifiers that we used in this study which makes it suitable for real-time applications. This classifier is widely used by [53,54,52] for face and skin detection purposes.

**Support Vector Machine (SVM)** introduced by Vapnik in [55] is another commonly used classifier for face and skin detection applications and applied by many researchers including[56,57,58]. SVM is a two-class classifier aimed to find the hyper plane which separate two classes with maximum marginal space between them. This study uses the same parameters for SVM as Khan et al. in [18]. We have used polynomials up to exponent three to construct the kernel. Complexity parameter is set to 1, tolerance parameter is set to 0.001 and epsilon for round-off error is set to 10^−12^.

The input feature vector for all classifiers includes 3 attributes which resemble three color components in the color space. Training and testing sub-datasets are constructed by dividing each dataset into two distinct complementary subsets with ratio of 75 percent for training sub-sets and 25 percent for the testing sub-sets. More details on training and testing datasets are summarized in Table 2.

**Table 2:** Training and testing datasets statistics.

Dataset	Subset	No. Images	Skin Pixels	Non-skin Pixels
Dataset A (899 images)	Training set	674	~ 29 million	~ 112.3 million
	Test set	225	~ 9.7 million	~ 37.5 million
Dataset B (400 images)	Training set	300	~ 5.2 million	~ 25.5 million
	Test set	100	~ 1.8 million	~ 8.5 million
Dataset C (1118 images)	Training set	838	~ 289.5 million	~ 718.5 million
	Test set	280	~ 96.5 million	~ 239.5 million

Classifiers have been trained using the entire training sub-set at each dataset. The skin pixels were taken from the manually segmented face and skin regions in training sub-set images while the non-skin pixels were taken from the complement of these images. While this study employed 10 fold stratified cross validation techniques to assess the accuracy of predictive model on the training sub-sets, we only report the evaluation results on the testing subsets. For testing, the trained classifier probes each image from the testing sub-set individually and generates a *skin binary mask* for each image. Each mask was compared at pixel level with corresponding skin segmented image from *ground truth* and generates a confusion matrix including the prediction outcomes and the actual values. Performance measures including FPR, Precision, TPR and F-score are driven from the confusion matrix. Skin detection performance is measured by averaging the performance measures of the individual images in the testing sub-set.

### Evaluation Metrics

This study uses True Positive Rate (TPR), False Positive Rate (FPR), Precision and F-Score to evaluate the performance of classifiers. TPR which refers to detection ratio is defined as (6):

$$\text{TPR}=\frac{{\text{N}}_{\text{TP}}}{{\text{N}}_{\text{S}}}$$ (6)

where N_TP_ is the number of correctly detected skin pixels and N_S_ is the total number of skin pixels. FPR refers to false alarm ratio given by (7):

$$\text{FPR}=\frac{{\text{N}}_{\text{FP}}}{{\text{N}}_{\text{NS}}}$$ (7)

where N_FP_ is the number of non-skin pixels which were falsely identified as skin pixel and N_NS_ is total number of non-skin pixels. F-score is harmonic mean of precision and recall values defined as (8):

$${\text{F}}_{\text{Score}}=\frac{2\;\text{TP}}{2\;\text{TP}+\text{FP}+\text{FN}}$$ (8)

Precision is another evaluation metric that we report in our experiments. It is the proportion of true positives against all positive results and defined as (9):

$$\text{precision}=\frac{\text{true positive}}{\text{true positive}\;+\;\text{false positive}}$$ (9)

Receiver Operating Characteristic (ROC) curves plots the True Positive Rate on Y axis against the False Positive Rate on X axis at various threshold settings. This study used ROC curves to give a visual perception about the proposed color space performance on skin detection.

### Experimental Results and Analysis

This section is aimed to measure skin detection performance using the proposed *SKN* color space. Three datasets including Dataset A, Dataset B and Dataset C in conjunction with four classifiers including Naïve Bayes, Random Forest, SVM and MLP were used to carry out this experiment. In this experiment classifiers have been trained using the entire training sub-sets of each dataset. The evaluation was also performed using all testing sub-sets of each dataset. Dataset statistics including the number of training and testing images are explained in Table 2. Classifiers parameters were explained in Experimental Setup section. Table 3 summarizes the skin detection evaluation metrics including FPR, Precision, TPR and F-score.

**Table 3:** Skin detection performance measures obtained using the proposed *SKN* color space. Significant results are in bold.

	Dataset A (HGR) Training set: 674 images / Testing set: 225 images				Dataset B (ECU) / Training set: 300 images / Testing set: 100 images				Dataset C (AR & FERET) / Training set: 838 images / Testing set: 280 images			
	FPR	Precision	TPR	F-1	FPR	Precision	TPR	F-1	FPR	Precision	TPR	F-1
**Naïve Bayes**	0.080	0.921	0.920	0.920	0.082	0.912	0.908	0.909	0.075	0.919	0.915	0.915
**Random Forest**	**0.037**	**0.964**	**0.964**	**0.964**	0.061	0.944	0.944	0.944	**0.042**	**0.958**	**0.958**	**0.958**
**SVM**	0.037	0.963	0.963	0.963	**0.059**	**0.945**	**0.945**	**0.945**	0.071	0.921	0.915	0.916
**MLP**	0.052	0.945	0.943	0.943	0.079	0.926	0.926	0.926	0.112	0.880	0.865	0.866

Experiments showed that the proposed hybrid *SKN* color space has produced significant average TPR and F-scores of 0.953 and FPR of 0.0482 using the Random Forest Classifier. The proposed color space produced its best performance in experiment on Dataset A. This might be due to high level of contrast and low dynamic range in Dataset A images which ease skin segmentation task. On the other hand, a slight drop in proposed color space performance was observed in experiments on Dataset B. This might be due to wide range of skin type, uncontrolled lighting condition and presence of skin-like colors in images from Dataset B which challenge the skin segmentation.

From the classifier perspective, Random Forest with average F-score and Precision rate of 0.953 and FPR of 0.0482 has the best performance among all classifiers in this study. SVM classifier with average F-score and Precision rate of 0.941 and FPR of 0.057 marginally underperformed as compared to Random Forest Classifier. On the other hand Naïve Bayes with average F-score rate of 0.912 and Precision rate of 0.915 and FPR of 0.080 deliver the poorest results among all classifiers in this study. MLP classifier generates relatively similar average results as Naïve Bayes classifier. Even though our training and testing data includes both bad and well exposed images, classifiers have mostly been trained with well exposed images so it is predictable that the performance of the proposed method drops when it comes to badly exposed images. Although, variance in results among three datasets is inevitable, Table 3 shows this variance is relatively insignificant (for example TPR and F-score of Random Forest classifier have variance of 0.02). This implies the robustness of the proposed color space to a wide range of input images.

**Fig 10:**
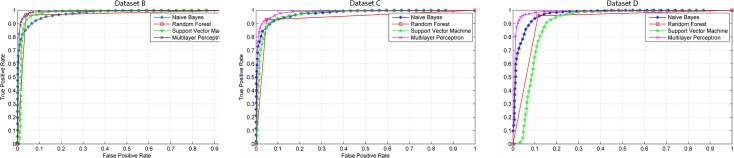
ROC curves of skin detection using the proposed *SKN* color space obtained from Datasets A B and C.

Fig 10 shows the ROC curves of each dataset obtained using the proposed *SKN* color space. The ROC curves show the tradeoff between FPR and TPR at various thresholds. Naïve Bayes, Random Forest, SVM and MLP classifiers were used to generate the curves. Apart from the SVM classifier which only uses one threshold value, we used 50 values of classification threshold at range of [0, 1]. For each threshold, average TPR and FPR have been measured using the images in the testing subsets. Visual inspection on ROC curves in Fig 10 roughly implies the superiority of the Random Forest over the other classifiers. However for more accurate comparison, Area Under Curve (AUC) of ROC curves in Table 4 is used to compare the performance of the proposed color space under different classifiers. Table 4 shows Random Forest with average AUC of 0.984 yields superior results compared to Naïve Bayes, SVM and MLP Classifiers. We can conclude that Random Forest classifier and proposed *SKN* color space are a perfect match for skin detection applications.

**Table 4:** AUC of skin detection using the proposed *SKN* color space obtained from Datasets A B and C.

	Naïve Bayes	Random Forest	SVM	MLP
**Dataset A—Training set: 674 images / Testing set: 225 images**	0.977	0.989	0.963	0.976
**Dataset B—Training set: 300 images / Testing set: 100 images**	0.976	0.982	0.943	0.969
**Dataset C—Training set: 838 images / Testing set: 280 images**	0.971	0.985	0.922	0.903

In terms of memory consumption, Naïve Bayes classifier which uses probabilistic technique occupies the largest amount of memory compared with other classifiers in this study. Random Forest MLP and SVM have relatively similar memory consumption. In terms of elapsed time, our experiments show that Naïve Bayes is the fastest classifier in both training and testing phases. This might be due to simplicity of arithmetic operation in probabilistic techniques. Random Forest classifier also has relatively fast response time. On the other hand SVM and MLP classifiers are extremely slow especially in training phase. Despite the fact that memory consumption and elapsed time are subjective and machine dependent matters, they can still give a rough estimation of the classifier efficiency.

Table 5 presents the comparison of the proposed *SKN* color space with some existing color spaces including YUV, HSV, CIELAB, nRGB, RGB, YCbCr and YCgCr. These color spaces are frequently used for skin and face detection applications. Color space transformation formulas of these color spaces can be found in S1 Table. FPR, TPR and F-score are the evaluation metrics used in this comparison. Three classifiers including Naïve Bayes, Random Forest and SVM were used to carry out this experiment. To make this comparison fair, we have used identical classifier parameters and training/testing sub-sets for all color spaces. Classifier parameters are explained in Experimental Setup section. The classifiers are trained and tested using the parameters mentioned in Table 2. This comparison tells us which color space delivers better skin detection performance under identical parameters and circumstances.

**Table 5:** Comparison of the skin detection performance between the proposed *SKN* color space and some existing color spaces. Significant results are in bold.

Color Space	Classifier	Dataset A (HGR)-Training set: 674 images/Testing set: 225 images			Dataset B (ECU)-Training set: 300 images/Testing set: 100 images			Dataset C (AR & FERET)-Training set: 838 images/Testing set: 280 images		
		FPR	TPR	F-score	FPR	TPR	F-score	FPR	TPR	F-score
**SKN—(Proposed)**	Naïve Bayes	0.080	0.920	0.920	0.086	0.906	0.907	0.075	0.915	0.915
	Random Forest	**0.037**	**0.964**	**0.964**	**0.053**	**0.948**	**0.948**	**0.042**	**0.958**	**0.958**
	SVM	0.037	0.963	0.963	0.062	0.941	0.941	0.071	0.915	0.916
RGB	Naïve Bayes	0.156	0.873	0.870	0.489	0.647	0.560	0.189	0.833	0.829
	Random Forest	0.093	0.904	0.904	0.152	0.812	0.810	0.102	0.896	0.896
	SVM	0.104	0.888	0.889	0.180	0.799	0.800	0.142	0.883	0.881
HSV	Naïve Bayes	0.202	0.721	0.712	0.144	0.818	0.818	0.119	0.842	0.843
	Random Forest	0.066	0.919	0.920	0.112	0.863	0.863	0.077	0.913	0.913
	SVM	0.089	0.881	0.882	0.119	0.862	0.863	0.104	0.900	0.900
YUV	Naïve Bayes	0.132	0.893	0.892	0.122	0.842	0.844	0.152	0.849	0.849
	Random Forest	0.073	0.929	0.929	**0.123**	**0.880**	**0.880**	0.109	0.903	0.902
	SVM	0.077	0.922	0.922	0.114	0.871	0.872	0.123	0.899	0.899
YC_b_C_r_	Naïve Bayes	0.121	0.846	0.846	0.127	0.829	0.829	0.125	0.840	0.840
	Random Forest	**0.066**	**0.931**	**0.931**	**0.113**	**0.880**	**0.881**	**0.062**	**0.930**	**0.931**
	SVM	0.086	0.905	0.905	0.144	0.873	0.872	0.091	0.902	0.902
YC_g_C_r_	Naïve Bayes	0.135	0.835	0.835	0.143	0.829	0.829	0.186	0.820	0.820
	Random Forest	0.094	0.901	0.901	0.107	0.856	0.856	0.147	0.878	0.876
	SVM	0.102	0.889	0.890	0.169	0.851	0.852	0.118	0.855	0.856
nRGB	Naïve Bayes	0.170	0.820	0.820	0.214	0.783	0.787	0.190	0.816	0.816
	Random Forest	0.133	0.871	0.871	0.133	0.846	0.847	0.138	0.856	0.857
	SVM	0.141	0.866	0.865	0.152	0.843	0.844	0.147	0.852	0.852
CIELAB	Naïve Bayes	0.164	0.841	0.842	0.258	0.804	0.790	0.180	0.831	0.831
	Random Forest	0.111	0.894	0.893	0.154	0.845	0.845	0.116	0.887	0.887
	SVM	0.120	0.888	0.889	0.169	0.837	0.838	0.128	0.879	0.879

According to the experimental results in Table 5, the proposed *SKN* color space outperformed the existing color spaces in terms of FPR, TPR and F-score across all datasets used in this experiment. The best results in terms of TPR (0.964) was achieved by the proposed color space using Random Forest classifier over images from Dataset A. In terms of FPR once again the proposed color space with FPR of 0.037 using Random Forest classifier has the lowest false detection rate.

As can be observed, there is a clear decrease in performance of the color spaces in the results from Dataset B. This might be due to a wide range of diversity in skin tones, lighting conditions and presence of skin-like surfaces in images from this dataset. However the proposed color space with TPR of 0.948 and FPR of 0.053 in Dataset B remained relatively unaffected by the challenging scenario in Dataset B. We can infer that the proposed color space relatively mitigates the major challenges in skin detection such as variation of skin tone and diversity in lighting condition. On the other hand, an increase in performance of all color spaces can be observed in the results from Dataset A. This can be due to low dynamic range and relatively plain backgrounds in images from Dataset A. Results from facial images in Dataset C show that once again the proposed *SKN* color space outperformed other color spaces in this comparison. Hence, the proposed color space might bring some benefits to face detection applications. Apart from the proposed color space, our experiments show that YUV and YCbCr color spaces deliver relatively promising results in skin detection across all datasets in this comparison. Studies in [28,18,59,52,60] also support this result. On the other hand, our experiment shows that RGB color space has the worst skin detection performance among all color spaces in this comparison. Poor skin detection performance in RGB can be due to high amount of correlation among its components. Studies in [61,18] also addressed this issue.

From the classifier perspective, it can be observed that Random Forest classifier consistently outperformed Naïve Bayes and SVM in the majority of the measures in this comparison. The main reason for this might be due to presence of outlier data (under exposed or over exposed skin area) in skin dataset which significantly reduces the SVM performance. However it seems that Random Forest which uses bagging technique has been less affected by outlier data. Furthermore, large number of training instances significantly improves Random Forest performance while SVM classifier performance is not strongly dependent on the number of instances. Studies by Tan et al. [62] and Khan et al. [18] also show the decent performance of Random Forest in skin and face detection applications. SVM classifier which marginally underperformed compared to Random forest is the second best classifier in this comparison; however this classifier is considerably slower than Naïve Bayes and Random Forest. Naïve Bayes classifier generates the poorest results among other classifiers in this comparison. However it’s high processing speed makes it a desirable choice for real-time applications.

**Fig 11:**
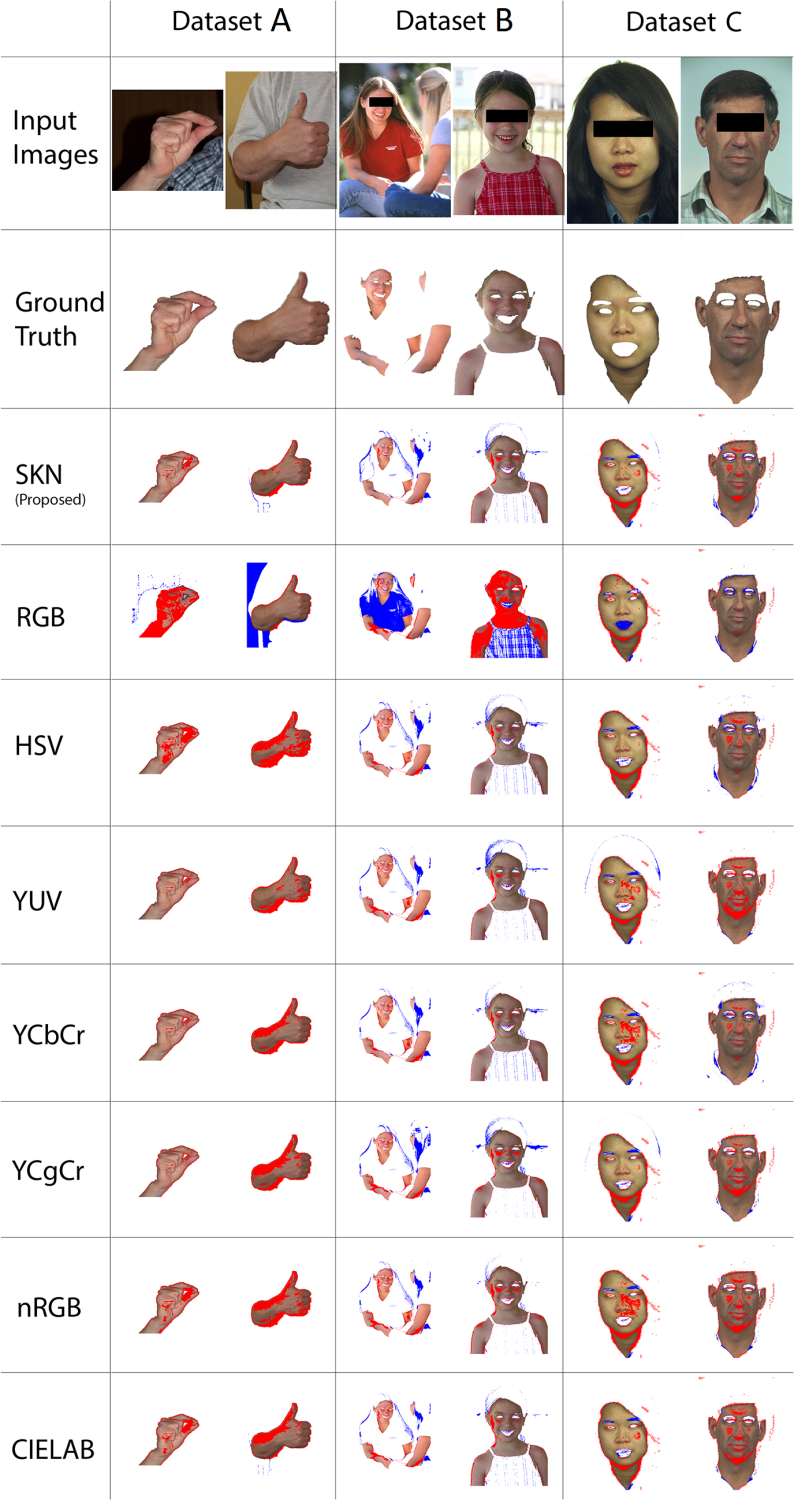
Qualitative comparison of the skin detection performance between the proposed *SKN* and some existing color spaces. Ground truth and original images are provided as reference.

Fig 11 shows the qualitative comparison between the proposed *SKN* color space and some existing color spaces including YUV, HSV, CIELAB, nRGB, RGB, YCbCr and YCgCr. For this comparison, two sample images are randomly chosen from the testing sub-set of each dataset. Random forest classifier was used to generate the skin mask for each image. To make this comparison fair, we have used identical classifier parameters for all color spaces. Random Forest parameters are explained in Experimental Setup section. Each image was compared at pixel level with corresponding skin segmented image from *ground truth*. Results are annotated with different color coding to increase its understandability and readability. Correctly detected skin regions (True Positive) are shown with actual skin colors. *Red* indicates False Negative, *Blue* indicates False Positive and True Negative are shown in *White*. It can be seen that the proposed *SKN* color space improved the skin detection accuracy compared with the existing color spaces. For the majority of the images in Fig 11, the proposed color space has lower average false positive rate (Blue) and False Negative rate (Red). YCbCr color space also shows relatively good performance in this comparison. RGB color space with relatively high False Positive (Blue) and high False Negative (Red) has the poorest results in this comparison. False detection usually happens due to the presence of skin-like colored objects like wood, furniture, sand etc. Pixel wise skin detection techniques are unable to properly differentiate skin-like colored objects. However applying an auxiliary technique like texture detection can dramatically reduce the false detection rate. On the other hand, issues like inappropriate lighting condition, extreme skin colors, and shade may increase the False Negative rate. Preprocessing techniques like color balancing, contrast adjustment, white balancing and color constancy techniques can reduce the False Negative rate.

## Conclusion

In this paper we have proposed a new hybrid color space. It is achieved by applying GA heuristic and PCA technique to 17 existing color spaces including HSI, HSV, LAB, LUV, nRGB, RGB, TSL, XYZ, YCbCr, YCgCr, YES, YIQ, YPbPr, YUV i1i2i3, RIQ and YQCr. GA heuristic searches for the optimal color component combination setups in terms of skin detection accuracy while PCA projects the GA optimal solution into a lower dimensional space. The proposed color space has been termed *SKN* (taken from word “Skin”) where “*S*” resembles the 1^st^ Principal Component, “*K*” denotes the 2^nd^ Principal Component and “*N*” indicates the 3^rd^ Principal Component of the GA optimal solution. Four classifiers including Naïve Bayes, Random Forest, SVM and Multilayer Perceptron have been used to measure and compare the performance of the proposed color space in terms of skin detection. Our experiments showed that the proposed hybrid color space improved skin detection accuracy compared with some existing color spaces. The results also indicate that among the classifiers we have used in this study, Random Forest is the most suitable classifier for skin detection. The proposed color space can be used in wide range of skin detection applications ranging from face detection, tracking body parts and hand gesture analysis, to retrieval and blocking objectionable content. Theoretically, the method that we have employed to produce our hybrid color space can be applied to any other image segmentation problems as long as enough training samples are provided.

## Supporting Information

S1 File Dataset access guideline. (DOCX)

S1 Table Color spaces transformation Formulas. (DOCX)
