# Supplementary material for: A Hybrid Color Space for Skin Detection Using Genetic Algorithm Heuristic Search and Principal Component Analysis Technique
Source: PLoS One. 2015 Aug 12;10(8):e0134828. doi: 10.1371/journal.pone.0134828 (PMC4534136; doi:10.1371/journal.pone.0134828)
Supplement: S1 File — (DOCX) [file pone.0134828.s001.docx]

**S1 file. Dataset access guideline**

The followings provide a guideline to access the Datasets and repository information that used in this study.

In order to access Dataset A (HGR) please follow the instructions provided by the original creator in the following link:

http://sun.aei.polsl.pl/~mkawulok/gestures/

Dataset B (ECU) also can be downloaded upon official request to Dr Lam Phung, the co-author of the (1) at: [phung@uow.edu.au](mailto:phung@uow.edu.au)

Dataset C (AR & FERET) can be accessed online upon official request to the original creators at following links:

http://www.nist.gov/itl/iad/ig/colorferet.cfm

http://www2.ece.ohio-state.edu/~aleix/ARdatabase.html

References

1. Phung SL, Bouzerdoum A, Chai D. Skin segmentation using color pixel classification: analysis and comparison. IEEE Trans Pattern Anal Mach Intell [Internet]. 2005 Jan;27(1):148–54.
