# Supplementary material for: A Hybrid Color Space for Skin Detection Using Genetic Algorithm Heuristic Search and Principal Component Analysis Technique
Source: PLoS One. 2015 Aug 12;10(8):e0134828. doi: 10.1371/journal.pone.0134828 (PMC4534136; doi:10.1371/journal.pone.0134828)
Supplement: S1 Table — (DOCX) [file pone.0134828.s002.docx]

**S1 Table. Color spaces transformation Formulas**

| **No#** | **Color Space** | **Transformation Formula** | **Description** |
| --- | --- | --- | --- |
| **1** | **RGB** | ----- | RGB color space is the basic and most used color space in digital image processing. |
| **2** | **nRGB** | $\left[ \begin{matrix} r \\ g \\ b \end{matrix} \right]= \left[ \begin{matrix} \frac{1}{R+G+B} & 0 & 0 \\ 0 & \frac{1}{R+G+B} & 0 \\ 0 & 0 & \frac{1}{R+G+B} \end{matrix} \right] \left[ \begin{matrix} R \\ G \\ B \end{matrix} \right]$ | Normalized RGB color spaces aimed to mitigate the dependency of the Luminance and chrominance components. |
| **3** | **XYZ** | $\left[ \begin{matrix} X \\ Y \\ Z \end{matrix} \right]= \left[ \begin{matrix} 0.6 & 0.174 & 0.2 \\ 0.3 & 0.59 & 0.114 \\ 0 & 0.066 & 0.117 \end{matrix} \right] \left[ \begin{matrix} R \\ G \\ B \end{matrix} \right]$ | XYZ is designed based on the response curves of the three color receptors of the human eyes |
| **4** | **YCbCr** | $\left[ \begin{matrix} Y \\ \mathrm{Cb} \\ \mathrm{Cr} \end{matrix} \right]=\left[ \begin{matrix} \\ 16 \\ 128 \\ 128 \end{matrix} \right]+\left[ \begin{matrix} 65.48 & 128.55 & 24.96 \\ -37.79 & -74.20 & 112 \\ 112 & -93.78 & -18.21 \end{matrix} \right] \left[ \begin{matrix} R \\ G \\ B \end{matrix} \right]$ | YCbCr is designed as a digital approach to handle video information in color television transmission systems. |
| **5** | **YUV** | $\left[ \begin{matrix} Y \\ U \\ V \end{matrix} \right]= \left[ \begin{matrix} 3 & 0.587 & 0.114 \\ -0.147 & -0.288 & 0.435 \\ 0.614 & -0.5148 & -0.1 \end{matrix} \right] \left[ \begin{matrix} R \\ G \\ B \end{matrix} \right]$ | YUV is used by PAL (Phase Alternation Line), NTSC (National Television System Committee) television signal transmission standards as an analogue approach to handle video information |
| **6** | **YIQ** | $I=V \cos33-U\sin33$  $Q=V\sin33+U\cos33$  $\left[ \begin{matrix} Y \\ I \\ Q \end{matrix} \right]= \left[ \begin{matrix} 3 & 0.587 & 0.114 \\ 0.596 & -0.274 & -0.32 \\ 0.21 & -0.523 & 0.312 \end{matrix} \right] \left[ \begin{matrix} R \\ G \\ B \end{matrix} \right]$ | YIQ color space is optionally used by NTSC composite color video standard for television signal transmission. YIQ is very similar to YUV color space. The only difference is the chrominance component which is angled by 33 degree. |
| **7** | **i1i2i3** | $\left[ \begin{matrix} i1 \\ i2 \\ i3 \end{matrix} \right]= \left[ \begin{matrix} 1/3 & 1/3 & 1/3 \\ 1/2 & 0 & -1/2 \\ -1/2 & 1 & -1/2 \end{matrix} \right] \left[ \begin{matrix} R \\ G \\ B \end{matrix} \right]$ | i1i2i3 Color space can be obtained through *Karhunen Loeve* transformation from the RGB color component. This color space Benefits minimal correlation among its components which makes it a suitable color space for image segmentation purposes especially for face detection. |
| **8** | **YES** | $\left[ \begin{matrix} Y \\ E \\ S \end{matrix} \right]= \left[ \begin{matrix} 0.253 & 0.684 & 0.063 \\ 0.5 & -0.5 & 0 \\ 0.25 & 0.25 & -0.5 \end{matrix} \right] \left[ \begin{matrix} R \\ G \\ B \end{matrix} \right]$ | YES is another linear color space in which used by many researchers in face and skin detection area. Y represents the luminance while E and S denote the chrominance of the color. |
| **9** | **HSV** | $H= \cos^{-1} \frac{\frac{1}{2}\left( \left( R-G \right)+\left( R-B \right) \right)}{\sqrt{\left( R-G \right)^{2}+\left( R-B \right)\left( G-B \right)}}$  $S=1-3\frac{\min\left( R,G,B \right)}{R+G+B}$  $V= \frac{1}{3} \left( R+G+B \right)$ | HSV is a perceptual color space which describes the colors with intuitive values (Hue, saturation and intensity). |
| **10** | **HSI** | $H= \frac{\pi}{2}-\tan^{-1} \left\{ \frac{2R-G-B}{\sqrt{3(G-B)}} \right\}$  $I=\frac{R+G+B}{3}$  $S=1-\frac{\min\left( R,G,B \right)}{I}$ | HSI is another perceptual color space which provides a more human like color description. |
| **11** | **TSL** | $T=\left\{ \begin{aligned} \frac{1}{2\pi}\tan^{-1} \frac{\acute{r}}{\acute{g}}+\frac{1}{4} \acute{g}<0 \\ \frac{1}{2\pi}\tan^{-1} \frac{\acute{r}}{\acute{g}}+\frac{3}{4} \acute{g}<0 \\ 0 \acute{g}=0 \end{aligned} \right.$  $S=\sqrt{\frac{9}{5}(\acute{r}^{2}+\acute{g}^{2})}$  $L=0.30R+0.59G+0.11B$ | TSL (Tint, Saturation, Lightness) color space was developed primarily for the purpose of face detection. |
| **12** | **Lab** | $L=116 f (\frac{Y}{Y_{0}})$  $a=500 (f \left( \frac{X}{X_{0}} \right)-f \left( \frac{Y}{Y_{0}} \right))$  $b=$ $500 (f \left( \frac{Y}{Y_{0}} \right)-f \left( \frac{Z}{Z_{0}} \right))$  Where X, Y and Z are defined in color space number 2 and X_0_=Y_0_=Z_0_=255 | Lab is based on CIE XYZ, attempts to linearize the perceptibility of unit vector color differences. |
| **13** | **Luv** | $L=116 f (\frac{Y}{Y_{0}})$  $u=13 L (\acute{u}-\acute{u}_{White})$  $v=13 L (\acute{v}-\acute{v}_{White})$  $\acute{u}=\frac{4X}{X+15Y+3Z}$  $\acute{v}=\frac{9Y}{X+15Y+3Z}$  Where X, Y and Z are defined in color space number 2 and X_0_=Y_0_=Z_0_=255 | Luv is a nonlinear reversible color space by CIE attempts to linearize the perceptibility of unit vector color differences. |
| **14** | **YPbPr** | $\left[ \begin{matrix} Y \\ P_{b} \\ P_{r} \end{matrix} \right]= \left[ \begin{matrix} 0.299 & 0.587 & 0.114 \\ -0.169 & -0.331 & 0.500 \\ 0.500 & -0.419 & -0.081 \end{matrix} \right] \left[ \begin{matrix} R \\ G \\ B \end{matrix} \right]$ | YPbPr is a space of color used in the electronic video. It is the analogical version of the color space YCbCr. |
| **15** | **YCgCr** | $\left[ \begin{matrix} Y \\ \mathrm{Cg} \\ \mathrm{Cr} \end{matrix} \right]=\left[ \begin{matrix} \\ 16 \\ 128 \\ 128 \end{matrix} \right]+\left[ \begin{matrix} 65.48 & 128.55 & 24.96 \\ -81.08 & 112 & -30.91 \\ 112 & -93.78 & -18.21 \end{matrix} \right] \left[ \begin{matrix} R \\ G \\ B \end{matrix} \right]$ | YCgCr color space designed for face detection purposes. similar to the YCbCr color space, it differs in the use of the Cg color component in- stead of the Cb one |
| **16** | **RIQ** | R= R  I = 0.596 R – 0.274 G – 0.32B  Q = 0.21 R – 0.523G + 0.312B | Hybrid color space based on R component from RGB color space and chromatic component of IQ from YIQ build for face recognition purposes. |
| **17** | **YQCr** | Y = 3R + 0.587 G + 0.114 B  Q = 0.21 R – 0.523G +0.312B  Cr = 128 + 112R - 93.78G - 18.21B | Hybrid color space based on Cr component from YCbCr and YQ components from YIQ color space. This color space is designed for face detection purposes. |
